# Tumor markers as an entry for SARS‐CoV‐2 infection?

**DOI:** 10.1111/febs.15499

**Published:** 2020-08-28

**Authors:** Pu Xia, Anna Dubrovska

**Affiliations:** ^1^ OncoRay ‐ National Center for Radiation Research in Oncology Faculty of Medicine University Hospital Carl Gustav Carus Technische Universität Dresden Helmholtz‐Zentrum Dresden‐Rossendorf Dresden Germany; ^2^ Institute of Radiooncology – OncoRay Helmholtz‐Zentrum Dresden‐Rossendorf (HZDR) Dresden Germany; ^3^ German Cancer Consortium (DKTK), Partner Site Dresden Dresden Germany; ^4^ German Cancer Research Center (DKFZ) Heidelberg Germany; ^5^ National Center for Tumor Diseases (NCT) Partner Site Dresden Dresden Germany

**Keywords:** Basigin, CD147, COVID‐19, Emmprin, SARS‐CoV‐2, spike protein

## Abstract

Coronavirus disease 2019 (COVID‐19), the highly contagious illness caused by a novel severe acute respiratory syndrome coronavirus 2 (SARS‐CoV‐2), has spread across the globe, becoming one of the most challenging public health crisis of our times. SARS‐CoV‐2 can cause severe disease associated with multiple organ damage. Cancer patients have a higher risk of SARS‐CoV‐2 infection and death. While the virus uses angiotensin‐converting enzyme 2 (ACE2) as the primary entry receptor, the recent experimental and clinical findings suggest that some tumor markers, including CD147 (basigin), can provide an additional entry for SARS‐CoV‐2 infection through binding to the viral spike (S) protein. In the absence of specific viral drugs, blocking of CD147 might be a way to prevent virus invasion. Identifying other target proteins is of high importance as targeting the alternative receptors for SARS‐CoV‐2 might open up a promising avenue for the treatment of COVID‐19 patients, including those who have cancer.

## Graphical abstract

Cancer patients are at higher risk of infection by a novel strain of coronavirus SARS‐CoV‐2, which causes coronavirus disease COVID‐19. Tumor cells overexpress potential SARS‐CoV‐2 receptors, including the primary viral receptor ACE2. Recent evidence suggests that some tumor markers, including CD147 (basigin), can also provide an entry point for SARS‐CoV‐2 through binding to the viral spike (S) protein. The blockade of CD147 could provide a way of preventing SARS‐CoV‐2 in the absence of specific viral drugs.

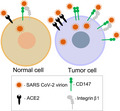

**Abbreviations**
ACE2: angiotensin‐converting enzyme 2
BSG: basigin
CD147: cluster of differentiation 147
COVID‐19: coronavirus disease 2019
CyPA: cyclophilin A
SARS‐CoV‐2: severe acute respiratory syndrome coronavirus 2

## Introduction

Coronavirus disease 2019 (COVID‐19), the illness caused by a novel severe acute respiratory syndrome coronavirus 2 (SARS‐CoV‐2), has spread across the world, becoming one of the leading causes of death in some places [1, 2]. The clinical manifestation of COVID‐19 infection includes pneumonia, respiratory illness, gastrointestinal and neurological symptoms, and multiple organ damage [3, 4, 5, 6, 7]. In addition to the direct invasion, the immune hyperactivation substantially contributes to the COVID‐19 toxicity [7, 8]. As the number of COVID‐19 infections surpassed 15 million globally and more than 600 000 infected people have died, according to the World Health Organization (WHO) report [9], scientists are struggling to understand the viral tropism better and develop the therapeutic approaches for preventing virus infection.

Cancer patients belong to a high‐risk group for SARS‐CoV‐2 infection, having a more severe course of the disease and a high case fatality rate [10, 11, 12, 13, 14]. It can be partially attributed to the older age and the weakened immune system of cancer patients as a consequence of treatment and the immunosuppressive tumor effect [13, 14]. However, no conclusive evidence is yet available explaining the susceptibility of the oncology patients to SARS‐CoV‐2 infection.

## Invasion by SARS‐CoV‐2 and tumor marker expression: causality or coincidence?

The SARS‐CoV‐2 belongs to β‐coronavirus genera and contains a single‐stranded, positive‐sense RNA genome encoding four structural proteins, including spike (S), nucleocapsid (N), membrane (M) and envelope (E), and several accessory proteins. Coronavirus spike (S) protein mediates the receptor binding of the cell surface and induces the fusion of viral and cellular membranes followed by viral genome entry into human target cells [15]. Analyses of the SARS‐CoV‐2 genome indicated that the virus uses angiotensin‐converting enzyme 2 (ACE2) as the entry receptor [16] that was later confirmed by functional studies of SARS‐CoV‐2 infection in the hACE2 transgenic mice models [17]. Single‐cell RNA sequencing provided evidence that multiple organs with high ACE2 expression, including respiratory tract, lung, esophagus, liver, colon, heart, kidney, ileum, rectum, and stomach, might be vulnerable to SARS‐CoV‐2 infection [18].

**Fig. 1:**
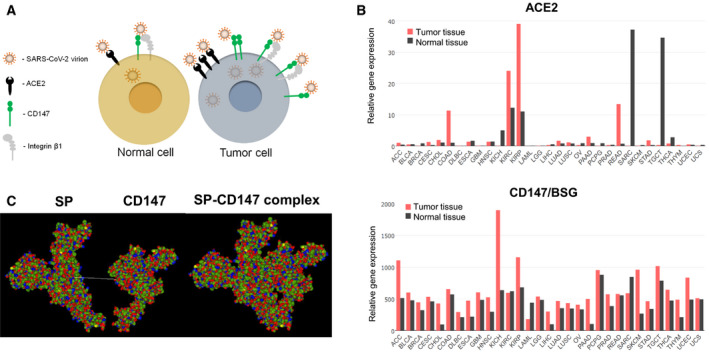
(A) Tumor cells overexpress potential SARS‐CoV‐2 receptors. (B) The gene expression profile across all tumor samples and paired normal tissues. The data are obtained using Gene Expression Profiling Interactive Analysis based on The Cancer Genome Atlas (TCGA) data. Relative gene expression is shown as transcripts per kilobase million (TPM). TCGA Study Abbreviations: ACC, adrenocortical carcinoma; BLCA, bladder urothelial carcinoma; BRCA, breast invasive carcinoma; CESC, cervical squamous cell carcinoma and endocervical adenocarcinoma; CHOL, cholangiocarcinoma; COAD, colon adenocarcinoma; DLBC, lymphoid neoplasm diffuse large B‐cell lymphoma; ESCA, esophageal carcinoma; GBM, glioblastoma multiforme; HNSC, head and neck squamous cell carcinoma; KICH, kidney chromophobe; KIRC, kidney renal clear cell carcinoma; KIRP, kidney renal papillary cell carcinoma; LAML, acute myeloid leukemia; LGG, brain lower grade glioma; LIHC, liver hepatocellular carcinoma; OV, ovarian serous cystadenocarcinoma; PAAD, pancreatic adenocarcinoma; PCPG, pheochromocytoma and paraganglioma; PRAD, prostate adenocarcinoma; READ, rectum adenocarcinoma; SARC, sarcoma; SKCM, skin cutaneous melanoma; STAD, stomach adenocarcinoma; TGCT, testicular germ cell tumors; THCA, thyroid carcinoma; THYM, thymoma; UCEC, uterine corpus endometrial carcinoma; UCS, uterine carcinosarcoma. (C) Predicting the complex of the host cell protein CD147 and coronavirus spike protein (SP) using the Hex 8.0 docking approach. BSG protein structure was downloaded from RCSB Protein Data Bank (PDB code: 3B5H). Based on homologous modeling, a PDB formatted file containing the predicted structure of coronavirus SP was generated by phyre2 software (http://www.sbg.bio.ic.ac.uk/phyre2) [26]. The tentative BSG‐SP docking was displayed using the HEX 8.0 program [27, 28]. 3D model of BSG and SP interaction was analyzed by HEX 8.0 with fast Fourier transform (FFT) algorithm using the following parameters: Correlation type—Shape + Electro+DRAS; FFT Mode—3D; Post‐processing—none; Grid dimension—0.6; Solutions—2000; Receptor range—180; Ligand range—180; Twist range—360; Distance range—40.

Of note, several types of malignant tumors have high ACE2 expression levels, including colon, kidney, pancreatic, and rectal cancer, which can potentially suggest a higher COVID‐19 prevalence for patients with these types of malignancies (Fig. 1A,B). The ACE2 protein was found to be highly expressed in lung adenocarcinoma (LUAD) and lung squamous cell carcinoma (LUSC) [19] that can be associated with a high susceptibility of lung cancer patients to SARS‐CoV‐2 Infection. On the other hand, cells expressing low ACE2, such as immune cells, can also be potentially infected by SARS‐CoV‐2 as it was shown for SARS‐CoV, suggesting that other receptors may facilitate the virus entry [20, 21]. In support of this hypothesis, recent studies showed that another cell surface protein CD147 could potentially bind to SARS‐CoV‐2 spike protein and serve as an additional infection route [16] (Fig. 1A,C).

Cluster of differentiation 147 (CD147) also termed basigin (BSG), or extracellular matrix metalloproteinase inducer, is a transmembrane glycoprotein with pleiotropic functions that is overexpressed in a broad range of malignant tumors and serves as a promising prognostic biomarker for glioblastoma, colorectal, oral, lung, prostate, and many other types of cancer [22]. CD147 has a substantially higher transcription level in both normal and tumor tissues than ACE2 (Fig. 1B). Furthermore, CD147 plays a role in the inflammatory response as a receptor for cyclophilin A (CyPA), a potent chemotactic factor for inflammatory leukocytes and activator of the intracellular antiviral response [22]. The CD147‐interacting partners, such as integrins, which are also abundant in many types of cancer, are additional candidate receptors for SARS‐CoV‐2 entry. Still, experimental proof for this hypothesis is required [23] (Fig. 1A,B).

Cluster of differentiation 147 represents a promising target for anticancer therapy. Treatment of unresectable hepatocellular carcinoma with ^131^I‐labeled CD147‐specific antibody metuximab (Licartin) combined with transcatheter arterial chemoembolization showed promising safety and efficacy (NCT00829465). The safety and clinical activity of CD147‐targeting CAR‐T treatment are currently analyzed in phase I clinical trials for patients with recurrent glioblastoma (NCT04045847) and advanced hepatocellular carcinoma (NCT03993743).

A study of Wang *et al*. posted on BIORxiv showed that CD147 could provide a new entry for SARS‐CoV‐2 infection. *In vitro* experiments showed that blocking of CD147 on the surface of African green monkey kidney cells (Vero E6) by meplazumab, a humanized anti‐CD147 antibody inhibited SARC‐CoV2 replication [16]. According to the preprint report on clinical trial study NCT04275245, meplazumab improved the outcome of patients with COVID‐19‐associated pneumonia with a favorable safety profile [24]. Of importance, meplazumab does not only target the virus replication but also attenuates T‐cell chemotaxis induced by CyPA and therefore inhibits COVID‐19‐associated inflammation [24].

However, more evidence is warranted to understand the possible correlation between the expression level of CD147 in cells or tissues and SARS‐CoV‐2 susceptibility. We also need to consider that the most lethal transmission route of SARS‐CoV‐2 is a lung and bronchial infection. In contrast, the viral concentration in blood is low [25], and the amount of viral particles that reach each organ through the blood is different. Moreover, in addition to the direct invasion, SARS‐CoV‐2 might damage multiple organs through collateral effects from cytokine overproduction and clotting.

At present, there is no effective treatment method for COVID‐19 patients except for a broad‐spectrum antiviral medication such as remdesivir (GS‐5734) (NCT04365725, NCT04292899). In the absence of specific drugs for SARS‐CoV‐2, humanized anti‐CD147 antibodies such as meplazumab can potentially function as antiviral drugs to block the CD147‐spike protein complex formation and prevent the virus from entering the host cell.

## Conclusions

All in all, the preliminary experimental and clinical findings suggest that targeting the alternative receptors for SARS‐CoV‐2 spike proteins might open up new treatment options for COVID‐19 disease, and therefore, identifying other target proteins is of high importance. The CD147‐targeting therapy showed promise in the clinical trial. Further large‐scale clinical studies are anticipated to validate it as a potential treatment of SARS‐CoV‐2‐infected patients, including those who have cancer.

## Conflict of interest

The authors declare no conflict of interest.

## Author contributions

PX and AD conceived of the presented idea. PX and AD analyzed the literature, wrote the paper, and approved the final version of the manuscript.
